# A Pilot Study on Integrating Videography and Environmental Microbial Sampling to Model Fecal Bacterial Exposures in Peri-Urban Tanzania

**DOI:** 10.1371/journal.pone.0136158

**Published:** 2015-08-21

**Authors:** Timothy R. Julian, Amy J. Pickering

**Affiliations:** 1 Department of Civil and Environmental Engineering, Stanford University, Stanford, CA, United States of America; 2 Woods Institute for the Environment, Stanford University, Stanford, CA, United States of America; Columbia University, UNITED STATES

## Abstract

Diarrheal diseases are a leading cause of under-five mortality and morbidity in sub-Saharan Africa. Quantitative exposure modeling provides opportunities to investigate the relative importance of fecal-oral transmission routes (*e*.*g*. hands, water, food) responsible for diarrheal disease. Modeling, however, requires accurate descriptions of individuals’ interactions with the environment (i.e., activity data). Such activity data are largely lacking for people in low-income settings. In the present study, we collected activity data and microbiological sampling data to develop a quantitative microbial exposure model for two female caretakers in peri-urban Tanzania. Activity data were combined with microbiological data of contacted surfaces and fomites (*e*.*g*. broom handle, soil, clothing) to develop example exposure profiles describing second-by-second estimates of fecal indicator bacteria (*E*. *coli* and enterococci) concentrations on the caretaker’s hands. The study demonstrates the application and utility of video activity data to quantify exposure factors for people in low-income countries and apply these factors to understand fecal contamination exposure pathways. This study provides both a methodological approach for the design and implementation of larger studies, and preliminary data suggesting contacts with dirt and sand may be important mechanisms of hand contamination. Increasing the scale of activity data collection and modeling to investigate individual-level exposure profiles within target populations for specific exposure scenarios would provide opportunities to identify the relative importance of fecal-oral disease transmission routes.

## Introduction

Diarrheal diseases caused by exposure to pathogenic agents are a leading cause of under-five mortality and morbidity in sub-Saharan Africa [1]. In addition, exposure to non-pathogenic fecal bacteria may contribute to child morbidity associated with malnutrition and child stunting [2]. Despite the known and potential impacts of fecal exposures on child health, there is little understanding of the relative importance of fecal-oral disease transmission routes in developing countries [3]. Contemporary research largely focuses on transmission through drinking water and food. However, recent evidence has highlighted the role of non-dietary ingestion pathways (e.g., ingestion of soil and contaminants via hand-to-mouth contacts) in children’s microbial exposures in low income countries [3,4]. In one study, infants in peri-urban areas of Zimbabwe were observed consuming large quantities of fecal bacteria through ingestion of soil and chicken feces [5]. Other studies have demonstrated high concentrations of fecal bacteria on surfaces and soils in developing countries, suggesting these matrices may influence child exposure to fecal contamination [4,6].

Quantitative exposure and risk modeling provides opportunities to identify routes responsible for exposure to feces. Quantitative microbial risk assessment (QMRA) models identify risks associated with infectious diseases exposures [7]. Examples of QMRA models investigating non-dietary ingestion routes include quantifying risks from nosocomial surfaces, food preparation surfaces, and cleaning laundry [8–10]. Risk assessment models have been used to quantify risks associated with specific activities, understand relative contributions of various exposure pathways, highlight the need for quantification of exposure factors, and identify effective interventions [11,12]. For example, Mattioli et al. (2015) applied this framework to estimate that between 97–98% of total fecal matter ingested by a Tanzanian child is due to hand-to-mouth contact events as compared to consumption of contaminated drinking water [13]. One limitation to the accuracy and applicability of risk assessment models is the reliance on accurate descriptions of individuals’ interactions with the environment. Mattioli et al. (2015), for example, modeled Tanzanian children’s interactions with the environment based on data collected about children from the United States [13]. Traditionally, human-environment interaction data are collected by one or more of the following methods: activity recall, activity diaries, structured observation, and third person videography [14]. Videography is considered superior to other activity data collection methods because it is more accurate, eliminates recall bias, and provides an opportunity to record difficult-to-remember events, events of short duration (e.g., hand-to-mouth contacts), and specific sequences of events [14,15]. Third person videography, combined with video translation, has been used to quantify activity data in order to estimate child exposure to chemical contaminants [16,17]. The activity data provided by videography and videotranslation are known as micro-level activity time series data (MLATS). MLATS data are second-by-second sequences of discrete contact events. The high resolution data on sequential contacts of activity data can be applied to understanding dermal and non-dietary exposures. In this study, first person perspective videography (FPV) is employed. FPV shifts the position of the camera from a third person perspective to a first person perspective by using a small, portable, head-mounted videocamera. The videocamera is mounted with a forward and downward angle to capture the full range of motion of the participant’s hands. When correctly framed, the angle of the videocamera is able to capture all hand-to-mouth and object-to-mouth contacts. Although FPV has been previously employed in sociological research, the present study provides the first known instance of using FPV to collect contact data [18].

The study objective was to demonstrate the application and utility of second-by-second activity data collection to understand fecal contamination exposures on hands in the developing world. The objective is accomplished by piloting FPV to capture activity data to parameterize a quantitative model of fecal bacteria exposures. The study provides a framework for increasing the scale of the described methods (activity data collection and microbial exposure profile modeling) to identify the relative importance of fecal-oral disease transmission routes in low-income countries. Microbial exposure profiles are time series of microbial contamination concentrations on surfaces (for example, hands). The profiles are then used to identify factors influencing contamination and estimate subsequent risks of infection from interactions with the surface. By providing a framework for modeling individual-level exposure profiles, we provide insight into methods needed to better understand inter-individual variability in fecal-oral disease transmission.

## Materials And Methods

### Participant Recruitment

Two women in a low-income urban community within Dar Es Salaam, Tanzania were recruited coincident with an ongoing field trial of hand hygiene among female caretakers for children under five years old.

### Ethics Statement

The following protocol and consent procedures were approved by both the Stanford University Research Compliance Office for Human Subjects Research and the Muhimbili University’s Institutional Review Board. Verbal informed consent was obtained from both participants following the communication of an approved script in Swahili (the participants’ native language) by a trained field enumerator. Written consent was not necessary because the research fulfilled the Stanford University Human Research Protection Program policy that verbal consent is sufficient when the research presents no more than minimal risk of harm and does not involve any procedures which would otherwise require written consent if conducted outside of the research context. Research proceeded only following affirmation of both understanding and consent, according to the approved protocol.

### Videography

**Fig 1:**
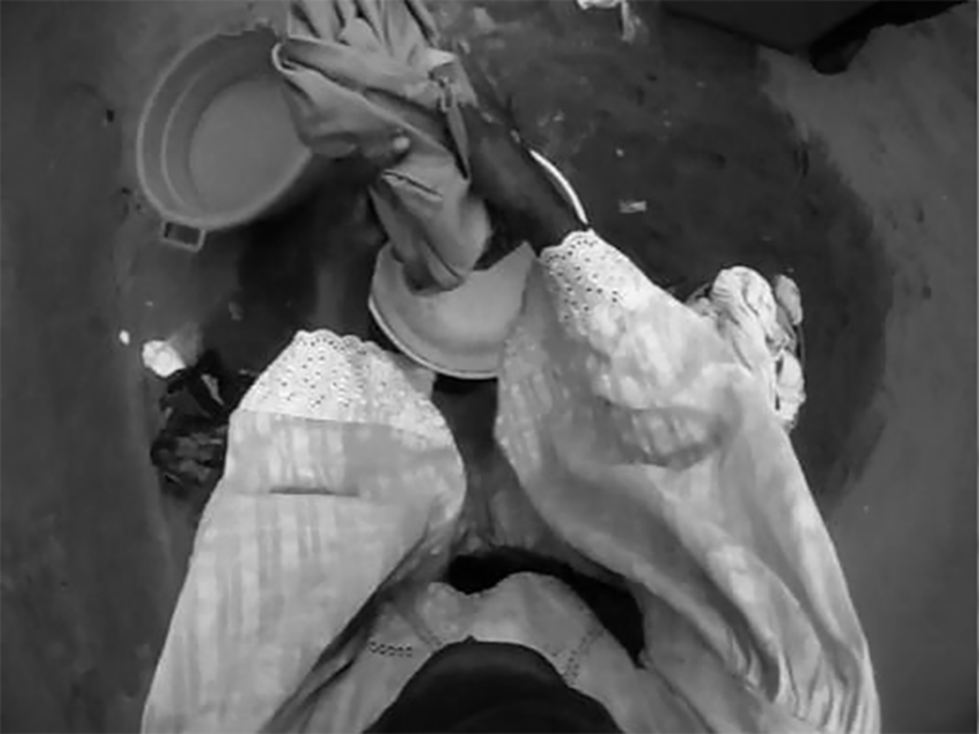
Field of view of video from Participant A, converted to grayscale.

Video cameras (GoPro Digital Hero 5, Woodman Labs, San Mateo, CA) were attached to head mounts such that the camera angled forward and downward to capture the majority of the hands’ range of motion (Fig 1). The two participants were instructed to complete their normal activities for two consecutive thirty-minute intervals from approximately 8:30 AM until 9:30 AM. In total, two hours of video were collected.

### Video-Translation

A modified version of Virtual Timing Device (VTD) Software (SamaSama Consulting, Sunnyvale, CA) was used to translate the videotape footage into MLATS for the caretakers’ right and left hands [14]. The VTD Software provides an interface consisting of two grids; each grid contains cells that represent various options for a) location of female caretaker, and 2) objects contacted by hand of caretaker. Each location and object is represented by a button on the software interface and can be specified by the user. The two locations in the first grid included: 1) inside and 2) outside. The second grid specified objects, which included surfaces that a hand contacted, water that a hand was immersed in (e.g., when washing clothing), and other special designations (e.g., the hand is not touching anything).

The objects that were specified for this study were chosen during an initial screening of the videos based on what objects were most frequently contacted. The eighteen surfaces in the objects grid included: 1) wooden broom, 2) metal bucket, 3) burlap sack, 4) charcoal, 5) clothing, 6) dirt or sand, 7) wooden door, 8) participant’s own face (face), 9) food scraps, 10) participant’s own hands, 11) money, 12) plastic objects, 13) metal cooking utensils including cooking pots and handles, 14) rubber, 15) bar soap, 16) stone, 17) wooden objects, and 18) paper towel used for hand sampling. The four water immersion objects included: 1) washing clothing, 2) washing hands, 3) water for drinking, and 4) water for hand sampling. The two special designations included: 1) nothing, and 2) not in view. To use VTD Software, a researcher views the video of the caretaker and in real time selects buttons for the location and object the participant is contacting. VTD records the sequence of the objects contacted along with the duration that the object is contacted. Contacts are recorded for all events where fingers, palm, and/or back of the hand come into direct contact with an object. Only one object can be contacted at a time, and when no object is contacted, the object category “nothing” is selected. The category “not in view” is selected when the hand is out of view and it is unclear what object the hand is contacting. Output from VTD is a computer text file with each line providing the location of the caretaker, the object contacted, and the duration of the contact. The present study provides data on the objects contacted by the left and right hands of the female caretakers. Data from VTD were imported into Microsoft Excel 2007 for analysis. As is common with video-translation using VTD, quality control measures are required (Beamer 2012). There was one researcher who translated the two hours of videotape. The same researcher then reviewed the tapes second-by-second to ensure the translations were accurate. When errors were identified, short segments (15 seconds to 2 minutes) were translated a second time, integrated into the data file to replace the corrected segment, and the file was reviewed again.

### Microbial Sampling

Hand samples were obtained from participants prior to collection of video. The hand samples were obtained using the hand rinse sampling method, which includes contacts with both water (characterized in the exposure model as the object ‘water used for hand sampling’) and a paper towel (characterized as the object ‘paper towel used for hand sampling’). [19]. In brief, the hand rinse method includes placing hands consecutively in a Whirl-pak bag (Nasco, Fort Atkinson, WI, USA) containing 350 ml of Uhaibottled drinking water pre-screened for the absence of target bacteria (i.e., *E*. *coli* and enterococci) and dosed with sodium thiosulfate. *E*. *coli* and enterococci were chosen as target bacteria because they are indicative of fecal contamination and co-occur with fecal pathogens on hands [19,20]. Additionally, both *E*. *coli* and enterococci are more abundant and easier to quantify than fecal pathogens. Both hands were placed into one bag, as opposed to measuring contamination on each hand individually, to decrease the lower limit of detection for bacterial contamination.

In addition to hand rinses, five fomites were sampled (see Table 1). Samples were chosen from fomites that participants had contacted during the video recording, as observed by the data collectors. The samples were collected to provide data on bacterial contamination of fomites contacted by the participants. Therefore, the samples were chosen as a representative subset of all fomites that participants had contacted during the video recording, as observed by the data collectors. Fomite samples were obtained by swabbing a surface of roughly 10 cm x 10 cm with polyester-tipped swabs pre-wet in 10 ml of ¼-strength Ringer’s solution (Oxoid Limited, Hampshire, UK) [4]. Hand rinse and fomite samples were transported on ice to the laboratory for analysis within 4 hours of sample collection.

**Table 1:** Concentrations of Bacteria (*E*. *coli* And Enterococci) on Objects Used in the Exposure Model.

		Microbiological Sampling^a^ ^,^ ^b^		Values Adjusted for Sampling Efficiency^a^ ^,^ ^c^		
Source	Fomite (Material)	*E*. *coli*	Enterococci	*E*. *coli*	Enterococci	Corresponding Object Categories
**Participant A**	Floor (*Soil*)	> 500	280	2500	1400	Dirt or Sand
	Broom (*Wood*)	7.5	< 2.5	37.5	12.5	Wooden Broom
	Bucket (*Plastic*)	2.5	< 2.5	12.5	12.5	Metal Bucket, Plastic Objects, Metal Utensils
	Bag (*Burlap*)	150	70	750	350	Burlap Sack
	Clothing (*Cloth*)	< 2.5	2.5	12.5	12.5	Clothing
**Participant B**	Plate (*Plastic*)	> 500	58	2500	290	Food Scraps
	Stool (*Wood*)	65	7.5	325	37.5	Wooden Door, Wooden Objects
	Tool Handle (*Rubber*)	< 2.5	< 2.5	12.5	12.5	Rubber
	Clothing (*Cloth*)	< 2.5	2.5	12.5	12.5	Clothing
	Bucket (*Plastic*)	70	10	350	50	Metal Bucket, Plastic Objects, Metal Utensils
**Assumptions** ^**d**^	Assumed Contaminated	DNM	DNM	12.5	12.5	Charcoal, Their Own Face, Money, Stone
	Assumed Clean	DNM	DNM	0	0	Not in View, Nothing, Paper Towels, Bar Soap, Washing Clothes, Washing Hands, Water Used for Drinking, Water Used for Hand Sampling

DNM, did not measure.

^a^Concentrations expressed in units of CFU/100 cm^2^ or CFU/100 ml.

^b^Refers to concentrations recovered from objects

^c^Refers to surface concentration used in the exposure model by adusting measurements to account for a sampling efficiency of 20%.

^d^Refers to object categories that were not similar to any fomites tested; for these objects either no contamination (0 CFU/100 cm^2^ or 0 CFU/100 ml) or the lower limit of detection (12.5) was assumed.

All samples were processed via membrane filtration to enumerate enterococci using mEI media per EPA method 1600 [21] and *E*. *coli* using MI media per EPA Method 1604 [22] as described elsewhere [3,19]. Lower and upper limits of detection for *E*. *coli* and enterococci on fomites are estimated at approximately 2.5 and 500 CFU/100 cm^2^. Limits of detection for bacteria on fomites were calculated using a countable range of 1–200 colonies following filtration of 2/5^ths^ (2 ml out of 5 ml) of Ringer’s solution used for fomite sampling. Lower and upper limits of detection for bacteria on hands are estimated at 3.5 and 700 CFU / 2 hands. Limits of detection on hands were calculated using a countable range of 1–200 colonies following filtration of 2/7^ths^ (100 ml out of 350 ml) of water used for hand sampling.

### Exposure Assessment Model

The exposure model is a modified version of the conceptual model for estimating microbial exposures from fomites previously described in Julian et al. (2009)[23]. Two exposure models were developed separately, one model for *E*. *coli* and one for enterococci contamination of hands. In brief, every object identified using videotranslation was assigned a quantitative estimate of surface *E*. *coli* and enterococci concentration and a quantitative estimate of the fraction of bacteria transferred on contact (Tables 1 and 2). Using the micro-level activity time series data, we assume the sequential contact of each object transferred bacteria to or from the hands based on the surface concentrations of the object and hand and the object-specific transfer efficiency consistent with the work of Julian et al. (2009) [23]. Similarly, we assumed parameters besides surface type, transfer direction, and magnitude did not influence transfer (e.g., wetness) [23]. We assumed every contact impacted the concentration of bacteria on the hand: bacteria transferred from hand-to-object when bacterial concentrations were greater on hands than objects and from object-to-hand when concentrations were greater on objects than hands [23]. The amount of bacteria transferred to or from the hand was determined by multiplying the concentration gradient between the hand and object by the transfer efficiency [23]:

$$C_{Hf}=C_{Hi}+T(C_{O}-C_{Hi})$$

Where *C*_*Hf*_ is the final concentration on the hand (CFU/100cm^2^), *C*_*Hi*_ is the initial concentration on the hand (CFU / 100cm^2^), *C*_*O*_ is the concentration on the object (CFU/100cm^2^), and *T* is the transfer efficiency for the object (%). The assumption of transfer from the surface with greater contamination to that with lesser contamination is based on previous modeling work as there are limited data on the impact of existing surface contamination on the magnitude or direction of transfer between two surfaces composed of different materials. The time series of *E*. *coli* and enterococci concentrations on the surfaces of the each hand, as determined by both sequential contact events and bacterial inactivation, is the output from the exposure model [23]. Bacteria concentrations on hands are then converted, using an estimate of sampling efficiency, to concentrations measured via the hand sampling method (see Surface Concentration).

**Table 2:** Fractional Transfer Efficiency Values Used in Exposure Model.

	Transfer Efficiency			
Object Category	*E*. *coli*	Enterococci	Fomite from Reference^a^	Reference
Broom	0.22	0.043	Laminate	Lopez et al. (2013)
Burlap	0.068	0.01	Cotton	Lopez et al. (2013)
Charcoal	0.073	0.039	Granite	Lopez et al. (2013)
Clothing	0.068	0.01	Cotton	Lopez et al. (2013)
Dirt/Sand	0.073	0.039	Granite	Lopez et al. (2013)
Door	0.22	0.043	Laminate	Lopez et al. (2013)
Face	0.34	0.41	Lip	Rusin et al. (2012)
Food Scraps	0.002	0.06	Hamburger	Rusin et al. (2012)
Hands	0.34	0.41	Lip	Rusin et al. (2012)
Metal Bucket	0.038	0.04	Stainless Steel	Lopez et al. (2013)
Metal Cooking Utensils	0.038	0.04	Stainless Steel	Lopez et al. (2013)
Money	0.0005	0.002	Paper Currency	Lopez et al. (2013)
Not in View	-	-	None	Assumed
Nothing	-	-	None	Assumed
Paper Towel	0.52	0.44	Hand Sampling	Pickering et al. (2010)
Plastic Objects	0.22	0.043	Laminate	Lopez et al. (2013)
Rubber	0.22	0.043	Laminate	Lopez et al. (2013)
Soap	-	-	None	Heinze et al. (1988)
Stone	0.073	0.039	Granite	Lopez et al. (2013)
Washing Clothes	0.68	0.44	Hand Washing	Pickering et al. (2010)
Washing Hands	0.68	0.44	Hand Washing	Pickering et al. (2010)
Water—Hand Sampling	0.52	0.44	Hand Sampling	Pickering et al. (2010)
Water for Drinking	0.68	0.44	Hand Washing	Pickering et al. (2010)
Wood Objects	0.22	0.043	Laminate	Lopez et al. (2013)

^a^Refers to the fomite used in the reference literature to determine transfer of Gram negative (*i*.*e*., *E*. *coli*) and Gram positive (i.e., enterococci) bacteria.

### Surface Concentration

Surface bacteria concentrations for each object category were estimated. The ten fomites that were sampled (Table 1 “Measured”) were used to estimate surface concentrations for eleven object categories (wooden broom, metal bucket, burlap sap, clothing, dirt or sand, wooden door, food scraps, plastic objects, metal cooking utensils, rubber, and wooden objects). Because *E*. *coli* recovery from surfaces using cotton-tipped swabs removes only an estimated 20% of bacteria [24], the measured concentrations were divided by a sampling efficiency to estimate the actual surface contamination (Table 1 “Modeled”), using:

$$F_{I}=\frac{F_{M}}{f_{F}}$$

Where *F*_*I*_ is the initial concentration of the fomite, *F*_*M*_ is the concentration of bacteria measured on the fomite, and *f*_F_ is the sampling efficiency for *E*. *coli* using swabs (Table 3). Concentrations of bacteria for five categories (not in view, nothing, paper towel used for hand sampling, bar soap, and water used for hand sampling) were set equal to 0 CFU/100 cm^2^ or 0 CFU/100 ml under the assumption the surfaces were clean. Notably, even when bar soap is contaminated with bacteria, there is no detectable bacteria transfer to hands on contact [25,26]. Concentrations of bacteria for three categories (washing clothes, washing hands, and water used for drinking) were set equal to 25 CFU/100ml and 200 CFU/100ml for *E*. *coli* and enterococci, respectively, based on mean concentrations of *E*. *coli* and fecal streptococci, a group which includes enterococci, in stored water in Tanzania [19]. The object category for ‘their own hands’ was assigned a concentration equal to the concentration predicted by the exposure model for the other hand at the time of contact. Concentrations for the remaining four categories (charcoal, their own face, money, and stone), for which we have no data, were assumed to be contaminated at the lower detection limit (2.5 CFU/100 cm^2^), corresponding to contamination of 12.5 CFU/100 cm^2^ when accounting for sampling efficiency (Table 1).

**Table 3:** Exposure Model Parameters, Values, and References.

		Participant A		Participant B		
Variable	Description	*E*. *coli*	Enterococci	*E*. *coli*	Enterococci	Reference
C_M_	Hand Concentration, Measured (CFU / 2 hands)	> 700	> 700	> 700	340	This study
C_I_	Hand Concentration, Initial for Model (CFU / 2 hands)	1346	1591	1346	773	This study
C_F_	Fomite Concentrations	**See Table 1**				
f_H_	Sampling Efficiency, Hands	0.52	0.44	0.52	0.44	Pickering et al. (2012)
f_F_	Sampling Efficiency, Fomites	0.2	0.2	0.2	0.2	Moore et al. (2007)
S_H_	Surface Area of Hands	440	440	440	440	USEPA (2011)
S_F_	Surface Area of Contact, Hand-to-Fomite	44	44	44	44	Assumed
S_W_	Surface Area of Contact, Hand-to-Water	440	440	440	440	Assumed
K	Inactivation Rate on Hands	-0.003	-0.00017	-0.003	-0.00017	Pinfold (1990)
TE	Transfer Efficiency	**See Table 2**				

### Transfer Efficiency

Transfer efficiencies of bacteria between hands and objects were based on a literature review of transfer efficiencies in high relative humidity (relative humidity in Dar es Salaam, Tanzania in July 2009 was between 60% and 90%, Table 2) [19,20,27–29]. The transfer rate of bacteria between hands and water when the hands are immersed in contaminated water is based on the findings of O’Toole et al. (2009) [27]. Transfer efficiency for hand washing is based on the findings of Pickering et al. (2010) and Pickering et al. (2011) for female caretakers in Tanzania [19,20]. Although transfer efficiency of bacteria between hands and surfaces has been shown to be influenced by the type of surfaces, inoculum size, and characteristics of the contact event (pressure, friction, wetness), we simplified the model by neglecting these characteristics in line with our previous work which suggested that model output is relatively insensitive to transfer efficiency magnitude when multiple contacts are modeled [23,30].

### Hand Concentration

The model assumed that bacterial contamination of hands is impacted by contact, with bacteria transferring from the surface (object or hand) with the higher concentration of bacteria to the surface (object or hand) with the lower concentration. However, only the concentrations of bacteria on hands were assumed to be impacted; the concentrations of objects were assumed to remain at the measured concentration. For hands, the initial concentrations were calculated based on the results of the hand sampling at time 0, and were adjusted based on hand sampling removal efficiencies of 52% for *E*. *coli* and 44% for enterococci [19]. Based on estimated removal efficiencies, initial hand concentrations were calculated using:

$$C_{I}=\frac{C_{M}}{f_{H}}$$

Where *C*_*I*_ is the initial concentration on hands (CFU/2 hands), *C*_*M*_ is the measured concentration on hands using the hand sampling method (CFU/2 hands), and *f*_H_ is the hand sampling removal efficiency. Concentrations are then adjusted to units of colony forming units (CFU) per cm^2^ based on average hand surface areas of 440 cm^2^ [31].

### Bacterial Inactivation

We also accounted for inactivation on hands consistent with the model previously reported by Julian et al. (2009) [23]. The log_10_ inactivation rate (*k*_*EC*_) for *E*. *coli* was assumed to be 3 x 10^−3^ 1/s, based on the findings of Pinfold (1990), where 99% of *E*. *coli* were inactivated on hands in 10 minutes [32]. A lower log_10_ inactivation rate (*k*_ENT_) for enterococci of 1.7 x 10^−4^ 1/s was assumed based on a 50% decrease in fecal streptococci in 30 minutes also reported in Pinfold (1990) [32].

### Surface Area

To account for surface area during contact events, we assumed that a contact event transferred only the portion of bacterial contamination on the object in direct contact with the hand. For object contacts, we assumed an average 44 cm^2^ of contact area (10% of 440 cm^2^, the total hand surface area). For water contacts, we assumed total hand submersion (440 cm^2^, or100% of total hand surface area) [33,34]. We assumed duration of contact did not influence the fraction of bacteria transferred, consistent with the work of Cohen-Hubal *et al*. who found duration did not impact chemical residue transfer on contact [35]. This assumption was extended to include immersion of hand with water: we assumed duration of immersion did not impact fraction of bacteria transferred. However, immediately after the transfer event, we assumed that the bacteria transferred were uniformly distributed over the hand, in line with the previous model [23]

### Non-dietary Ingestion

Non-dietary ingestion of fecal bacteria was modeled assuming that all hand-to-own face contact events resulted in ingestion of bacteria from hands. The amount of bacteria ingested was modeled based on previous work in Julian et al. (2009)[23]:

$$D=S_{H}{TE}_{HF}C_{H}$$

Where D is the dose of bacteria ingested, S_H_ is the surface area of the hand in contact with the mouth, TE_HF_ is the percentage of bacteria transferred from the hand to the mouth, and C_H_ is the concentration of bacteria on the hands. Similar to transfer of bacteria between surfaces, we again assume the surface area of hand-to-mouth contact events equals 10% of the total hand surface area. Other sources of fecal bacteria ingestion (such as mouth contacts with contaminated objects besides hands, ingestion of contaminated water, and ingestion of contaminated food) were not included in the model because these events did not occur during the observation.

## Results

### Micro-level Activity Time Series

Participant A was recorded for 54:16 min and spent her time sweeping and washing laundry (Table 4). Participant B was recorded for 63:16 min and spent her time making metallic oil lamps for resale (Table 4). The left hand for Participant A contacted 16 different object categories 200 times. The right hand for Participant A contacted 18 different object categories 208 times. The left and right hands for Participant B contacted 11 and 15 different objects, respectively, 271 and 368 times.

**Table 4:** Frequency and Duration of Hand Contacts with Objects.

	Participant A				Participant B			
	Left Hand		Right Hand		Left Hand		Right Hand	
Objects	Contacts^a^	Time^b^	Contacts^a^	Time^b^	Contacts^a^	Time^b^	Contacts^a^	Time^b^
Broom	40 (44)	14:40 (27%)	26 (29)	15:40 (29%)	1 (0.9)	0:02 (0.1%)	-	-
Metal Bucket	-	-	3 (3.3)	1:18 (2.4%)	181 (172)	36:52 (58%)	182 (173)	16:33 (26%)
Burlap	1 (1.1)	1:10 (2.2%)	3 (3.3)	0:22 (0.7%)	6 (5.7)	0:36 (0.9%)	6 (5.7)	0:29 (0.8%)
Charcoal	1 (1.1)	0:30 (0.9%)	2 (2.2)	0:42 (1.3%)	-	-	-	-
Clothing	48 (53)	3:25 (6.3%)	30 (33)	1:57 (3.6%)	43 (41)	2:45 (4.3%)	65 (62)	4:50 (7.6%)
Dirt or Sand	1 (1.1)	0:16 (0.5%)	2 (2.2)	0:29 (0.9%)	1 (0.9)	0:01 (0%)	4 (3.8)	0:12 (0.3%)
Door	-	-	-	-	-	-	1 (0.9)	0:01 (0%)
Face (Own)	4 (4.4)	0:21 (0.6%)	-	-	3 (2.8)	0:02 (0.1%)	3 (2.8)	0:02 (0.1%)
Food Scraps	-	-	2 (2.2)	1:07 (2.1%)	-	-	-	-
Hands (Own)	5 (5.5)	0:17 (0.5%)	6 (6.6)	0:17 (0.5%)	-	-	-	-
Money	1 (1.1)	0:5 (0.1%)	2 (2.2)	0:10 (0.3%)	-	-	2 (1.9)	0:04 (0.1%)
Not in View	3 (3.3)	0:13 (0.4%)	3 (3.3)	0:20 (0.6%)	52 (49)	8:26 (13%)	31 (29)	4:30 (7.1%)
Nothing^c^	198 (219)	10:23 (19%)	139 (154)	6:44 (12.4%)	267 (253)	10:08 (16%)	339 (321)	7:02 (11%)
Paper Towel	-	-	-	-	1 (0.9)	0:10 (0.3%)	1 (0.9)	0:10 (0.3%)
Plastic	-	-	-	-	16 (15)	1:26 (2.3%)	17 (16)	0:58 (1.5%)
Plastic Pot	18 (20)	2:26 (4.5%)	22 (24)	1:56 (3.6%)	-	-	2 (1.9)	0:20 (0.5%)
Metal Cooking Utensils	7 (7.7)	0:13 (0.4%)	4 (4.4)	0:10 (0.3%)	-	-	57 (54)	22:10 (35%)
Rubber	-	-	-	-	-	-	12 (11)	2:09 (3.4%)
Soap	1 (1.1)	0:20 (0.6%)	18 (20)	3:09 (5.8%)	-	-	-	-
Stone	1 (1.1)	0:01 (0%)	2 (2.2)	0:03 (0.1%)	9 (8.5)	1:56 (3.1%)	2 (1.9)	0:08 (0.2%)
Washing Clothes	65 (72)	19:09 (35%)	61 (67)	17:56 (33%)	-	-	-	-
Washing Hands (Own)	1 (1.1)	0:10 (0.3%)	17 (19)	0:31 (1%)	-	-	-	-
Water for Drinking	-	-	3 (3.3)	0:35 (1.1%)	-	-	-	-
Water—Hand Sampling	1 (1.1)	0:32 (1%)	1 (1.1)	0:27 (0.8%)	1 (0.9)	0:41 (1.1%)	1 (0.9)	0:58 (1.5%)
Wood	5 (5.5)	0:07 (0.2%)	4 (4.4)	0:23 (0.7%)	9 (8.5)	0:12 (0.3%)	13 (12)	2:41 (4.2%)
**TOTAL**	**401 (443)**	**54:16 (100%)**	**350 (387)**	**54:16 (100%)**	**590 (559)**	**63:16 (100%)**	**738 (700)**	**63:16 (100%)**

-, did not contact.

^a^Number of contacts recorded over duration of observation and adjusted to frequency of contacts per hour (in parentheses).

^b^Total duration of contact reported in minutes: seconds format and percentage of total time in contact with each category (in parentheses).

^c^No object was in contact with the hand

Both hands of both participants spent 10–20% of the total observed time not in contact with any objects or surfaces (see Table 4). Specifically, the left and right hands of Participant A were observed to spend 19% (10:23) and 12% (6:44), respectively, of the total observed time not contacting any object. Similarly, the left and right hands of Participant B were observed to spend 16% (10:08) and 11% (7:02), respectively, not contacting any object.

For the majority of time, both hands for both participants were in full view of the camera (see Table 4). However, full extension of the arm and misplacement of the camera on the head during the second half of the video for one participant (Participant B) resulted in the hands frequently falling outside of the camera’s field-of-view. Participant A’s left and right hands were not in view 3 times each for a total duration of 0:13 (0.4%) and 0:20 (0.6%), respectively. Conversely, Participant B’s left and right hands were not in view 52 and 31 times for a total duration of 8:26 (13%) and 4:30 (7.1%), respectively.

### Enterococci and *E*. *coli* on Hands and Fomites

The initial enterococci concentration on Participant B’s hands was 340CFU / 2 hands. The corresponding *E*. *coli* concentration was above the limit of detection (>700 CFU / 2 hands) and therefore assumed to be 700 CFU / 2 hands. For Participant A, both initial enterococci and *E*. *coli* concentration were above the detection limit (>700 CFU / 2 hands) and so were also assumed to be 700 CFU / 2 hands. Both *E*. *coli* and enterococci concentrations on fomites ranged from <2 to **>**500 CFU/100 cm^2^ (see Table 1).

Measured concentrations on both hands and fomites were adjusted for the exposure model to account for sampling efficiency (Table 3). Initial concentrations for bacteria on hands ranged from 773 to 1591 CFU per 2 hands, or 387 to 796 CFU per hand. Fomite concentrations used for the exposure model, after accounting for sampling efficiency, ranged between 0 and 2500 CFU/100cm^2^.

### Exposure Model

**Fig 2:**
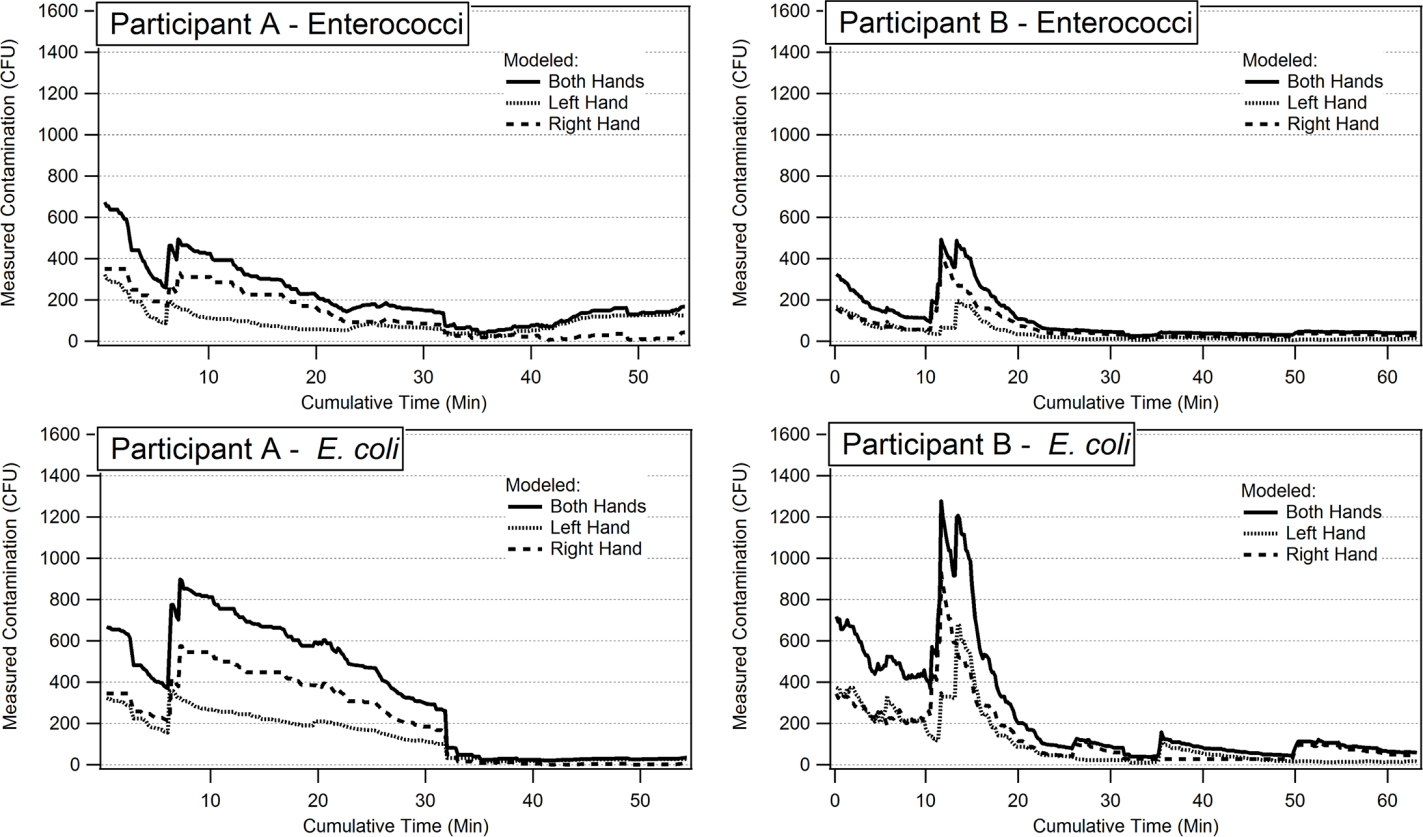
Modeled *E*. *coli* and enterococci concentrations on hands of participants, adjusted for sampling efficiency.

The modeled concentrations on the hands of each participant for *E*. *coli* and enterococci follow similar temporal trends. The concentration of bacteria increases substantially after 10 to 15 minutes, coincident with the participants’ contacts with dirt/sand (Fig 2). The bacteria concentrations then decrease. The decrease is more rapid for Participant B than for Participant A. The decreases are either due to contacts with surfaces with lower bacterial concentrations than on the hands (Participant A) or contacts with water (Participant B). The bacteria concentrations eventually reach levels near the detection limit and then remain low for both participants for the remainder of the study.

Fecal bacteria ingested is estimated based on hand-to-own face contact events. Participant A contacted her own face 4 times corresponding to an estimated cumulative ingestion of 46 CFU *E*. *coli* (ingestion events of 23, 22, 0.6, and 0.6 CFU *E*. *coli*) and 65 CFU enterococci (ingestion events of 33, 31, 0.6, and 0.7 CFU *E*. *coli*). Participant B contacted her own face 6 times corresponding to cumulative ingestion of 148 CFU *E*. *coli* (ingestion events of 24, 39, 18, 49, 16, and 3 CFU *E*. *coli*) and 38 CFU enterococci (ingestion events of 8, 14, 5, 8, 2, and 1 CFU enterococci).

## Discussion

Exposure to fecal contamination in the developing world is linked to poor child and maternal health [1,2]. In the present study, first person perspective videography is used to collect micro-level activity time series (MLATS) data and MLATS data are used to develop exposure models. The study highlights evidence that exposure to dirt or sand may be most responsible for contamination of caretaker’s hands and that water-related activities (e.g., washing clothing, washing hands) may not be effective at dramatically reducing *E*. *coli* contamination on hands. The impact of washing clothing, in particular, on *E*. *coli* concentrations may be due to *E*. *coli* contamination of laundry as observed in the United States and/or by use of soil or sand as a cleaning aid, as observed in Peru [36,37]. These findings further support the work of Pickering et al. (2011) that shows female caretakers’ activities can dramatically influence their hand contamination [20].

First person videography is capable of capturing MLATS for hands of study participants. When the camera is appropriately placed on the participant’s forehead, as was the case for Participant A, first person videography is able to capture more than 99% of all hand contacts. However, FPV is subject to more data loss than third person perspective videography when the camera is misplaced, as was the case for Participant B. Misalignment of the camera for Participant B impacted the entire hour duration resulting in substantial data loss: 13% of the total time for the left hand and 7% for the right hand. As a comparison, the time out of view for third person perspective videography for child exposure factors is approximately 9% (range of 2 to 18%) for the left hand and 7% (2–13%) for the right hand [38]. Two modifications would improve first person videography as a tool for collecting MLATS: a camera with a wider field of view (wide angle lens) and a headset that adjusts to the individual participant and reduces risk of misalignment. Two other concerns for FPV are distractions associated with wearing a camera on one’s head and invasions of personal privacy. Although neither participant appeared, nor described themselves as, distracted or concerned about privacy, a larger sample size of participants would be needed to understand these concerns. Future studies should include adaptation of protocols to allow for temporary removal of video cameras as well as survey-based data collection from participants on issues concerning FPV surveillance compliance.

Given the small sample size of two participants, generalizable conclusions drawn from the collected MLATS are limited. The potential range of activities for female caretakers in low-income countries is large, incorporating not only the activities observed but other domestic (e.g., shopping, cooking, water collection) and/or economically-productive (e.g., agricultural, commercial) activities. Nevertheless, similarities between the participants were observed, including high levels of activity characterized by frequent repeat contacts of common objects (e.g., metal bucket objects, metal cooking utensils, brooms, clothes) and infrequent contacts with rare objects (e.g., charcoal, dirt/sand, money, stone). This is noteworthy as the exposure models suggest that infrequent contacts (e.g, contact with dirt and sand in Fig 2, Participant B) were responsible for dramatic increases in bacterial contamination on hands. The reliance on a single video translation observer may have influenced categorization of objects contacted which were not validated via inter-observer, but accuracy of the frequency and duration of contact events was ensured via the described intra-observer validation protocol.

Estimates of parameter values strongly influence model outputs. The data used in the model were drawn from a combination of literature review, microbiological sampling, and videography. Of these sources, the microbiological sampling data are likely both the most influential and least certain. First, we did not collect estimates of microbial surface contamination for all of the fomites that the female caretakers contacted (e.g., charcoal, money, stone, faces). In the future, a wider array of surfaces should be sampled to ensure all surfaces contacted have an accurate estimate of microbial contamination. Second, bacteria concentrations on hands exceeded the upper limit of detection for the assays. We therefore had to assume that bacteria concentration on hands was equal to the upper limit of detection for the exposure models. This assumption directly influenced the performance of the model as our assumption that *E*. *coli* hand contamination was at the upper detection limit may have been an underestimate. Underestimating initial *E*. *coli* concentrations likely resulted in a model that systematically underestimated hand contamination for the remaining time series. In the future, assays should be designed to increase the upper limit of detection of hand contamination so that an accurate estimate of initial hand contamination can be used to initialize and investigate the accuracy of the model.

There are notable limitations to our work. One limitation is that we measured bacterial contamination on hands from both hands simultaneously instead of measuring bacterial contamination of each hand separately. Because the left and right hands are not necessarily equally contaminated, the initial concentrations for each hand may be biased. Having separate data points for each hand individually would have enabled comparison of modeled to estimated hand contamination for each hand. A second limitation is that our model is not validated. Although we attempted to validate the model by collecting hand samples at the middle and end of the videography (data not shown), the data suffered from limited interpretability. This was because only two additional samples were collected for each exposure profile, the samples represented contamination on both hands, and half of the measured concentrations were outside of the countable range. Future studies looking to validate exposure models should collect hand samples at a timescale relevant to expected changes in microbial contamination. Our model, for example, suggests dirt/sand contacts dramatically increase bacterial contamination.

Exposure models provide insight into the relative importance of contact events on microbial contamination of hands. The work presented here highlights the disproportional role on infectious disease exposures that infrequent contact events may have. Given the small sample size of this study, it is important to consider increasing the scale of activity data and microbial data collection. Applying the model to develop individual-level exposure profiles within target populations would provide opportunities to identify human-environment interactions that most influence fecal-oral disease transmission routes.
